# A Rare Case of Sinus of Valsalva Aneurysm Rupture Resulting From Infective Endocarditis and Requiring Surgery

**DOI:** 10.7759/cureus.81944

**Published:** 2025-04-09

**Authors:** Nozomu Kishio, Yasuhiro Ichikawa, Shun Kawai, Yusuke Nakano, Shigeo Watanabe, Masami Goda, Daisuke Machida

**Affiliations:** 1 Pediatric Medicine, Yokohama City University, Yokohama, JPN; 2 Pediatric Medicine, Fujisawa City Hospital, Fujisawa, JPN; 3 Pediatric Cardiology, Saiseikai Yokohamashi Tobu Hospital, Yokohama, JPN; 4 Pediatric Cardiology, Yokohama City University, Yokohama, JPN; 5 Cardiac Surgery, Yokohama City University, Yokohama, JPN

**Keywords:** atopic dermatitis, infective endocarditis, sinus of valsalva, staphylococcus aureus, transthoracic echocardiography

## Abstract

Infective endocarditis (IE) is a life-threatening infection. Although some cases with early diagnosis are treated with antibiotics only, others are associated with intracardiac complications and/or sepsis, necessitating intensive care and surgical repair. Congenital heart disease (CHD) and atopic dermatitis (AD) are risk factors for IE, with AD being a common comorbidity in children with CHD-related IE due to impaired skin barrier function. In the present case, the patient’s untreated AD compromised his skin integrity, serving as a portal for *Staphylococcus aureus* entry, which was subsequently isolated from blood cultures. We report a rare case of IE with sinus of Valsalva aneurysm rupture (SoVR) requiring surgical repair. A two-year-old boy with a ventricular septal defect and AD was referred to our hospital for IE with sepsis. Blood culture was positive for methicillin-susceptible *Staphylococcus aureus*. The patient entered the intensive care unit (ICU), and antibiotic administration was started. Transthoracic echocardiography (TTE) showed a vegetation and SoVR with perforation of the right coronary cusp, leading to an aorta-to-right ventricle shunt. On the 11th day after admission, TTE showed dilatation of the ruptured sinus of Valsalva and exacerbation of the aorta-to-right shunt. Surgical repair was performed on the same day. The postoperative course went well, and the patient was extubated on postoperative day (POD) five and discharged from the ICU on POD nine. TTE showed trivial aortic regurgitation and diminished aorta-to-right ventricle shunt. Antibiotics were discontinued one month after admission. The patient was discharged from our hospital on the 40th day after admission. In this case, the underlying AD likely contributed to the IE by serving as an entry point for *S. aureus*. Close follow-up and systemic management, always with surgical treatment in mind, were important for determining the timing of surgical intervention. Clinicians should also recognize the role of AD in increasing IE risk and emphasize proactive skin care for CHD patients to prevent severe infections.

## Introduction

Infective endocarditis (IE) is a severe bacterial infection associated with significant cardiac complications, particularly among patients with congenital heart disease (CHD). Sinus of Valsalva aneurysm rupture (SoVR) is an exceptionally uncommon yet life-threatening complication of IE, resulting from bacterial-induced weakening of the aortic sinus wall and subsequent rupture into adjacent cardiac chambers.

This study describes a unique pediatric case of IE complicated by right coronary sinus rupture into the right ventricle, the most common rupture site, in a two-year-old with an underlying ventricular septal defect (VSD) and atopic dermatitis (AD). This study further underscores the complexity of decision-making in pediatric IE with SoVR, particularly in relation to the optimal timing of surgical intervention. Additionally, this case uniquely suggests the potential contributory role of dermatologic conditions, such as untreated AD, as an entry point for bacterial pathogens in children with congenital heart defects.

Thus, this report aimed to contribute novel clinical insights by highlighting distinct management considerations and emphasizing the importance of multidisciplinary collaboration and close clinical and echocardiographic follow-up in pediatric IE cases complicated by sinus of Valsalva rupture.

## Case presentation

A two-year-old boy had a small ventricular septal VSD of the doubly committed juxta-arterial type, which had been found at birth at another hospital. He was followed up at the hospital afterwards, but as he had withdrawn from his outpatient visits, the details of the course of VSD are unknown. The patient developed a fever 10 days before admission to our hospital. On day six of illness, he was first evaluated at a general hospital due to persistent fever, elevated inflammatory markers (CRP 17.71 mg/dL, D-dimer 30.0 μg/mL), and thrombocytopenia (platelet count 9,000/μL). Due to concerns of an invasive bacterial infection, he was transferred to the intensive care unit (ICU) of another hospital. Broad-spectrum antibiotics (meropenem and vancomycin) were initiated. On day eight, blood cultures grew methicillin-susceptible *Staphylococcus aureus* (MSSA), and transthoracic echocardiography (TTE) identified a large vegetation above the aortic valve. He was diagnosed with IE and subsequently referred to our hospital for specialized management, given the severity of his condition. He was given transfusion of platelets for his thrombocytopenia. The patient was diagnosed with IE and was transferred to our hospital.

In addition to VSD, the patient also had untreated AD, which his father had also experienced. He also had a food allergy to milk and eggs. He had no dental caries. The patient’s physical examination showed Levine II/VI continuous murmur and pigmentation with AD. Laboratory tests showed the following: white blood cell count 25,600/μL, hemoglobin 8.3 g/dL; platelet count 34,000/μL; CRP 9.02 mg/dL; brain natriuretic peptide (BNP) 896.7 pg/mL; and D-dimer 14.9 μg/mL. TTE shows a large (18 x 14 mm) vegetation above the aortic valve in the right ventricular side, along with a sinus of Valsalva aneurysm rupture featuring right coronary cusp prolapse and an aorta-to-right ventricle shunt. Contrast-enhanced computed tomography revealed bilateral infiltrative shadows with cavities in the middle lobes, atelectasis in the lower lobes, consistent with pneumonia, pleural effusion, and a contrast defect in the spleen. His head magnetic resonance imaging showed no brain infarction.

**Figure 1:**
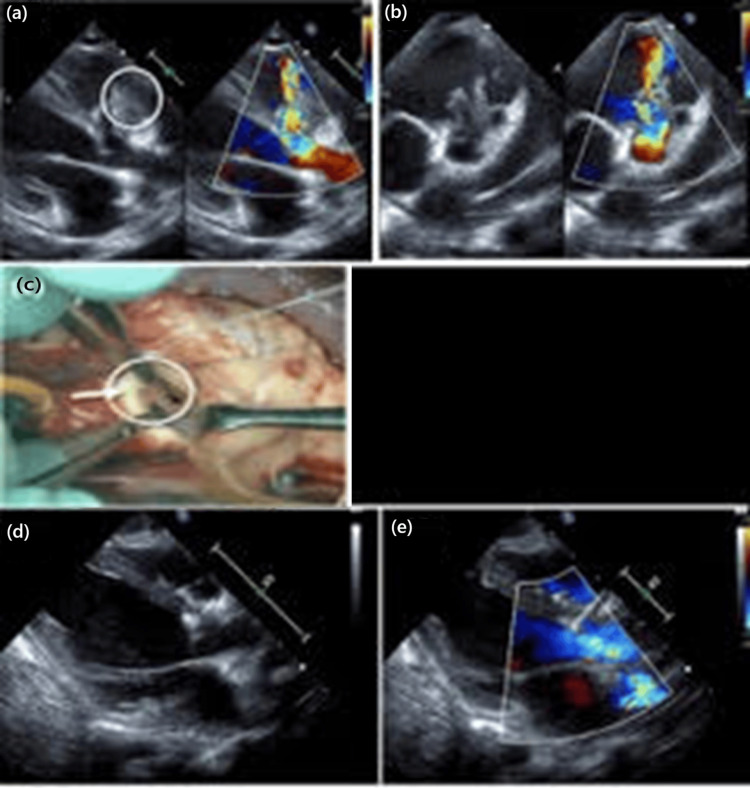
Serial echocardiogram and surgical findings. (a) Transthoracic echocardiogram (TTE) on the 11th day after admission in the long-axis view before surgery. TTE shows a large vegetation (white circle) above the aortic valve and sinus of Valsalva aneurysm rupture with right coronary cusp prolapse and left-to-right (L-R) shunt. (b) TTE on the 11th day after admission in the short-axis view with L-R shunt. (c) The surgical findings show severe destruction of the right cusp (white arrow) and a perforated aortic sinus of Valsalva (white circle). (d) TTE after surgery in the long-axis view. (e) TTE after surgery in the long-axis view, with closure of the rupture and diminished L-R shunt with trivial aortic regurgitation.

Mechanical ventilation was used for respiratory failure due to pneumonia. Catecholamine and diuretic administration and peritoneal dialysis were also necessary for circulation failure caused by sepsis. Transfusion of red blood cells, platelets, and fresh frozen plasma was required for anemia, thrombocytopenia, and coagulopathy due to disseminated intravascular coagulation. As blood culture was positive for MSSA, intravenous antibiotic administration was started with cefazolin (100 mg/kg/day) and gentamycin (7 mg/kg/day). Initially, the patient’s severe sepsis status was considered too critical to safely undergo immediate surgery. Therefore, our initial management strategy focused on stabilizing the patient through intensive antibiotic therapy and close monitoring. On day four of hospitalization, the patient was weaned from peritoneal dialysis and CRP was decreased. Frequent echocardiograms performed in the ICU revealed progressive dilatation of the ruptured sinus of Valsalva aneurysm and worsening of the aorta-to-right ventricle shunt, accompanied by destruction of the right coronary cusp and signs of poorly controlled heart failure (Figure 1, panels a, b). BNP was elevated (1342.6 pg/mL), and CRP (7.06 mg/dL), which had once decreased (0.9 mg/dL on day six), was elevated again. These echocardiographic findings on the 11th day after admission indicated impending hemodynamic compromise, prompting surgical intervention at that time. Surgery was performed, which showed severe right coronary cusp destruction, a perforated aortic sinus of Valsalva, and vegetation at the left ventricular tract (Figure 1, panel c). The patient underwent repair of the SoVR, VSD closure, aortoplasty, and vegetation debridement.

The postoperative course was uneventful. The CRP and BNP concentrations declined, and the patient was extubated on postoperative day (POD) five before being transferred from the ICU to the pediatric ward on POD nine. TTE showed trivial aortic regurgitation and diminished aorta-to-right ventricle shunt (Figure 1, panels d, e). In addition to an uneventful postoperative course, the Guidelines for the Prevention and Treatment of Infective Endocarditis recommend a treatment duration of four to six weeks from the negative blood culture [1]. Therefore, we felt that a four-week course of antibiotics from the negative blood culture on the first day of admission was sufficient, so the antibiotics were discontinued on the 31st day after admission. The patient had no fever and was discharged from our hospital on the 40th day after admission. As the patient also had uncontrolled AD, topical steroids were started during hospitalization and continued after discharge, and the patient was subsequently followed up by dermatologists for AD.

## Discussion

SoVR is a rare but severe complication of IE, particularly in pediatric patients. Previous retrospective studies reported SoVR in only two out of 216 IE cases [2] and four out of 20 pediatric IE cases, demonstrating its rarity [3]. We found only two previous pediatric reports of IE complicated by SoVR. Rotstein et al. described a two-year-old patient without known cardiac defects, infected by *Kingella kingae*, who developed acute heart failure due to rupture of the left sinus into the left atrium, requiring emergent surgical intervention [4]. Mukhopadhyay et al. reported a 16-year-old patient, also without known predispositions, who experienced rupture from the non-coronary sinus into the right atrium due to *Staphylococcus aureus* infection [5]. This patient was managed conservatively with antibiotics initially, followed by successful device closure. Compared to prior cases, this report is unique in several aspects. First, the presence of untreated AD likely played a pivotal role in facilitating *Staphylococcus aureus* entry into the bloodstream, leading to endocarditis. While CHD is an established risk factor for IE, the contribution of AD as a secondary risk factor has been underrecognized in pediatric populations. Given the impaired skin barrier function in AD, children with CHD may have an increased risk of developing severe infections, necessitating proactive dermatologic care as part of IE prevention strategies.

Another distinguishing feature of this case was the critical importance of frequent monitoring in guiding surgical timing. The initial TTE demonstrated a large vegetation above the aortic valve and aortic sinus rupture with right coronary cusp prolapse, leading to an aorta-to-right ventricle shunt. Although the patient was initially too unstable for surgery due to severe sepsis and multi-organ dysfunction, serial echocardiography showed progressive enlargement of the ruptured sinus and worsening left-to-right shunting, prompting urgent surgical intervention. In addition to imaging, laboratory markers such as persistently elevated BNP and worsening clinical signs of heart failure reinforced the need for early surgery. These findings underscore the importance of a multimodal approach - combining imaging, hemodynamic assessment, and systemic management - in determining the optimal timing for surgical repair in critically ill pediatric IE patients.

From a broader perspective, this case highlights the importance of dermatologic management in children with CHD to reduce the risk of IE. During hospitalization, topical steroid therapy was initiated, and the patient was referred for dermatologic follow-up post-discharge. This proactive approach is essential, as poorly controlled AD can serve as a persistent entry point for bacterial infections, increasing the risk of IE recurrence [6,7]. Pediatric cardiologists should incorporate skin care evaluation into routine CHD management, especially in patients with recurrent *Staphylococcus aureus* infections or a history of AD.

Regarding long-term outcomes, prior studies have reported favorable surgical results in pediatric SoVR cases, with no early postoperative deaths or aneurysm recurrence. However, potential complications such as late aortic regurgitation, residual shunting, and recurrent IE remain concerns. Our patient will require regular echocardiographic monitoring to assess valve function and early signs of recurrence. Additionally, strict adherence to IE prophylaxis guidelines, including optimal skin care and early infection management, will be critical in preventing future episodes.

## Conclusions

In conclusion, this study underscores the rare but serious complication of SoVR in a pediatric patient with IE and CHD, emphasizing the importance of early recognition and timely surgical intervention. The successful outcome was achieved through a multidisciplinary approach, integrating close echocardiographic monitoring, infection control, and surgical planning. Given the limited literature on IE complicated by SoVR in pediatric patients, this case provides valuable insights into its management and underscores the importance of considering skin barrier dysfunction as a potential contributor to severe infections in CHD patients.
